# Implementation, Mechanisms of Effect and Context of an Integrated Care Intervention for Vulnerable Families in Central Sydney Australia: A Research and Evaluation Protocol

**DOI:** 10.5334/ijic.4217

**Published:** 2019-07-25

**Authors:** John G. Eastwood, Susan Woolfenden, Erin Miller, Miranda Shaw, Pankaj Garg, Hueiming Liu, Denise E. De Souza, Roelof G. A. Ettema

**Affiliations:** 1 School of Women’s and Children’s Health, The University of New South Wales, Sydney, NSW, AU; 2 Ingham Institute of Applied Medical Research, Liverpool, NSW, AU; 3 Charles Perkins Centre, Menzies Centre for Health Policy, Discipline of Child and Adolescent Health, and School of Public Health, University of Sydney, NSW, AU; 4 Community Health Services, Sydney Local Health District, Level Camperdown, NSW, AU; 5 Sydney Institute for Women, Children and their Families, Camperdown, NSW, AU; 6 School of Humanities, Nanyang Technological University, SG; 7 The George Institute for Global Health, The University of New South Wales, AU; 8 Knowledge Centre Healthy and Sustainable Living, University of Applied Sciences, Utrecht, NL; 9 University Medical Centre, Utrecht, NL; 10 Discipline of Child and Adolescent Health, School of Medicine, University of Sydney, NSW, AU

**Keywords:** process evaluation, theory driven evaluation, critical realism, complex intervention, translational social epidemiology

## Abstract

**Introduction:**

In March 2014, the New South Wales (NSW) Government (Australia) announced the NSW Integrated Care Strategy. In response, a family-centred, population-based, integrated care initiative for vulnerable families and their children in Sydney, Australia was developed. The initiative was called *Healthy Homes and Neighbourhoods*. A realist translational social epidemiology programme of research and collaborative design is at the foundation of its evaluation.

**Theory and Method:**

The UK Medical Research Council (MRC) Framework for evaluating complex health interventions was adapted. This has four components, namely 1) development, 2) feasibility/piloting, 3) evaluation and 4) implementation. We adapted the Framework to include: critical realist, theory driven, and continuous improvement approaches. The modified Framework underpins this research and evaluation protocol for *Healthy Homes and Neighbourhoods*.

**Discussion:**

The NSW Health Monitoring and Evaluation Framework did not make provisions for assessment of the programme layers of context, or the effect of programme mechanism at each level. We therefore developed a multilevel approach that uses mixed-method research to examine not only outcomes, but also *what is working for whom and why*.

## Introduction

In March 2014, the New South Wales (NSW) State Government of Australia released the NSW Integrated Care Strategy to transform the delivery of care for patients, improve their health and wellbeing, and minimise costs associated with fragmentation of care delivery across the hospital and primary care sector. This was to be achieved by: “a) focusing on organising care to meet the needs of targeted patients and their carers, rather than organising services around provider structures; b) designing better connected models of health [and social] care to leverage available service providers to meet the needs of our smaller rural communities; c) improving the flow of information between hospitals, specialists, community and primary care providers; d) developing new ways of working across State government agencies and with Commonwealth funded programs to deliver better outcomes for identified communities; and e) providing greater access to out-of-hospital community-based care, to ensure patients receive care in the right place for them” [1].

The associated NSW Government’s Integrated Care Strategy funding enabled the establishment of an integrated care initiative called *Healthy Homes and Neighbourhoods (HHAN)*. The Initiative was designed as a population-based, family-centered, care-coordination network that functioned across agencies to assist vulnerable families to navigate the health and social care system, to keep themselves and their children safe, and in doing so, promote social cohesiveness [2]. The design was based on an earlier programme of research and collaborative design to support vulnerable families [3].

### The intervention

**Figure 1:**
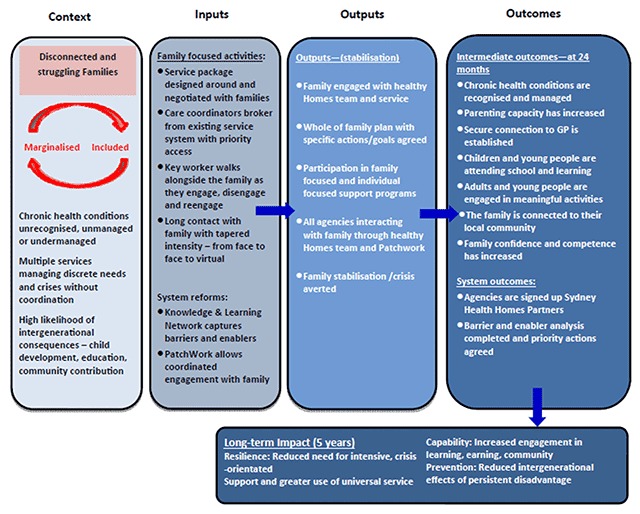
Theory of Change – HHAN Early Intervention and Clinical Elements.

HHAN is intended for vulnerable families with children unborn through to 17 years, whose complex health or social care needs impact on their ability to parent effectively and participate in community life. The intervention was designed to improve adult members’ participation in the social and economic life of the community through integrated management of their complex health and social conditions. In turn, the initiative benefits child members of the family by minimising the impact of adult complex health conditions on their safety, health, development and wellbeing. By employing a dyadic, or family-partnership approach, the intervention aims to interrupt complex intergenerational cycles of disadvantage, psychological trauma, underdeveloped parenting and associated poor health and development outcomes (Figure 1). The person-centred intervention is supported by other components that function at professional and organisational levels (see **Box 1**).

**Box 1: Healthy Homes and Neighbourhoods Key Features**

**Healthy Homes and Neighbourhoods**

The Healthy Homes and Neighbourhoods Integrated Care Initiative uses a stratified population-based approach to address the needs of families who are experiencing adversity, while supporting parallel interventions for families more generally. The approach to identifying the most vulnerable families who are disconnected from key services has been developed using existing perinatal risk-assessment systems, developing new cross-agency assessment and referral pathways, and improved hospital recognition of the needs of families using an e-health solution.

The initiative has the following key features:

1. Multiple core and non-core agencies **working together over a sustained period of time** (i.e. 5 years) with families with complex health and social needs
2. Co-design and co-production of the initiative in **partnership** with families and service partners
3. **All the needs of enrolled families are in scope** for the intervention, including housing, employment, income support and legal advice
4. An **early intervention and public health approach** to interrupting cycles of family disadvantage, poor health and psychological trauma
5. A **focus on efficiency** through the maximum use of, and leverage from, existing family, societal and government resources, including Medicare scheduled services
6. Use of **evidence-informed integrated care methods** by service partners, including family case conferencing, and ‘wrap-around’ care delivery
7. Encouraging families to have a **‘health home’** for all their health needs and supporting progress towards self-efficacy
8. **Providing a supporting structure to general practice** providers to care for families that are often seen to be ‘too difficult’
9. Development and implementation of **shared assessment tools and referral criteria**
10. Implementation of **family assessment and engagement tools** that can be used over the long-term to monitor the health and wellbeing of family members

A central element of the initiative is targeted long-term sustained cross agency care coordination. The design acknowledges the need for significant system redesign and commitment from partners. The initial model required a care coordination team with both project-funded and partner-funded components as a means of ensuring sustainable ‘collaboration’. The initiative also includes local elements through deliberate recruitment of families and service partnerships in the City of Canterbury and City of Sydney local government areas. This last component enabled the development of ‘demonstration-site’ place-based partnerships with local general practice, schools, family support agencies, local government, religious and faith-based organisations and community members.

### NSW Health Monitoring and Evaluation Framework

The programme of research which informs the development of the HHAN research and evaluation protocol is underpinned by the NSW Health Integrate Care Strategy Monitoring and Evaluation Framework [1]. That Framework proposes both formative and summative evaluations, detailed in Table 1.

**Table 1:** NSW Health Monitoring and Evaluation Approach [1].

Key steps	Outputs

Formative evaluation components	

**Discovery and planning through program logic**	Detailed program overview of activities and expected outcomes Key assumptions about how change will occur Anticipated outputs and outcomes
**Development of relevant indicators**	Process indicators and metrics recognising that both local and state-wide indicators exist Progressively develop new data collection mechanisms
**Development of purposeful road maps**	Develop road map milestones based on key evaluation questions emerging from the logic maps Develop milestones that reflect indicators, both qualitative and quantitative, that allow assessment of actual outcomes relative to expected outcomes
**Definition of key functional components of integrated care**	Common framework of functional components to facilitate the development and capture of core indicators
**Summative evaluation components**	

**Design of reporting approach**	Quarterly output/outcomes reports for discussion at local health district (LHD) performance meetings and integrated care governance committees Annual outcome evaluation reports
**Identification of data sources**	Identify all monitoring and evaluation data sources Use routine data collection wherever possible
**The philosophy of continuous improvement**	Continuous improvement strategy based on Plan-Do-Study-Act (PDSA) cycle approach Changes to program direction or arrangements based on reflection on monitoring results and outcome reports – what is working and what is not
**Detailed design and execution of evaluation approach**	Appropriateness, effectiveness, efficiency assessed at different stages of the program to determine immediate, intermediate and longer-term outcomes

The NSW Health Framework was updated in 2016 [4] to include state-wide approaches to identify integrated care cohorts that would enable tracking of patients across the continuum of care and assessing outcomes through health record linkage. The updated Framework also included a refined set of process indicators. The NSW Health Monitoring and Evaluation Framework is focused on the evaluation of the intervention theory with no explicit attempt to evaluate either context or programme mechanisms. Thus for the programme of research described below we will: 1) explicate implementation (intervention) theory as part of the NSW Health implementation evaluation and 2) examine programme theory using critical realist research and evaluation methods. Identifying contextual and programme mechanisms in the programme theory will be important for assessing the validity of claims made about what works, for whom, under what conditions, and why, and how what works or does not work may be attributed to the HHAN initiative.

### UK Medical Research Council (MRC) Framework

**Figure 2:**
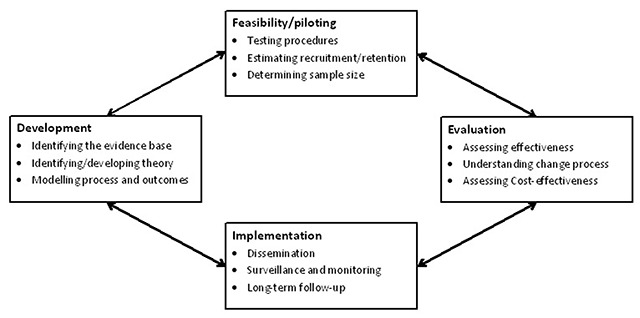
Key elements of the development and evaluation process [7].

Apart from adhering to the local standards recommended by the NSW Health Monitoring and Evaluation Framework [1], the research and evaluation framework for HHAN also aligns with recommendations proposed by the UK MRC Framework. The vast majority of care provided in the context of achieving integrated care is undertaken by care deliverers (professionals and informal caregivers) and includes multiple single interventions which interact with each other. That care is characterised as complex care or complex interventions [5]. The MRC model has proven to be a useful framework for developing, testing and implementing complex interventions [5]. As early as 2000, the UK MRC introduced a framework for evaluating complex interventions recommending sequential phases of development, feasibility testing and evaluation, culminating in the estimation of effect size via a randomised controlled trial [6]. The 2008 update provided a four-phase, cyclical framework of development, feasibility/piloting, evaluation, and implementation (Figure 2) [5].

A limitation of the MRC Framework as it stands, is that it relies only on independent verifiable observations. As a consequence, the MRC Framework does not allow for the essential inductive, and abductive, process required for developing complex interventions to fit context. Critical realist, and theory of change, research and design processes, however, enable theory driven design to be developed that takes historical and current context into account.

The research and evaluation protocol described here will draw on the NSW Health Integrated Care Strategy: Monitoring and Evaluation Framework [1,4], the 2008 UK Medical Research Council (MRC) Framework for complex interventions, and our previously reported critical realist methodology [8]. We have modified our previously reported critical realist methodology to better align with the 2008 MRC Framework. The methodology described here is designed specifically for an integrated care audience. As with all mixed-method research protocols it is also appropriate for the early section to have a strong methodological content.

The research and evaluation protocol is integrating a number of quite disparate and conflicting methodological approaches including critical realism, evaluation of complex interventions, theory of change, logic models and improvement science approaches. As such, we will introduce these in the next section, before describing our adapted evaluation framework and methods.

## Theory and Methods

### Critical Realism

As a contemporary philosophy of science, critical realism draws from both post-positivist and interpretivist traditions and views reality as an open system. It therefore acknowledges the fallibility of our understanding of reality. In keeping with post-positivist tradition, critical realists draw a distinction between the intransitive domain where reality exists independent of our knowledge of it, and the transitive domain which considers our generation of theories to derive incomplete understandings and knowledge about reality. Drawing on an interpretivist tradition, critical realists view the process of developing scientific knowledge and theory as socially constructed—political, historical and imperfect [9,10,11,12].

A second tenet in critical realist ontology proposes that three inter-related domains make up reality. These domains are: (1) the real—where entities are said to possess structures and mechanisms that have generative powers whether these are actualised or not; (2) the actual—where entities under certain conditions actualise the powers and mechanisms they possess to produce events, but that these may or may not be empirically observed; and (3) the observed or empirical—where entities actualize their powers and mechanisms under given conditions to produce events that are observed and experienced [10]. Thus, critical realism does not accept empirical observations as the only domain of reality that needs explanation. It seeks to include explanations about how entities are structured, their mechanisms and the conditions needed to activate those mechanisms.

### Theory driven approaches

We have also drawn here on the work of Blamey and Mackenzie who compared Theories of Change and Realist approaches to evaluation [13]. Put simply, Theory of Change (ToC) research focuses on intervention theory, while realist evaluation examines programme theory. Citing Weiss [14] they define “[intervention] theory” as “what is required to translate objectives into ongoing service delivery and programme operation” and “programme theory” as “the responses of the people to programme activities”.

**Figure 3:**
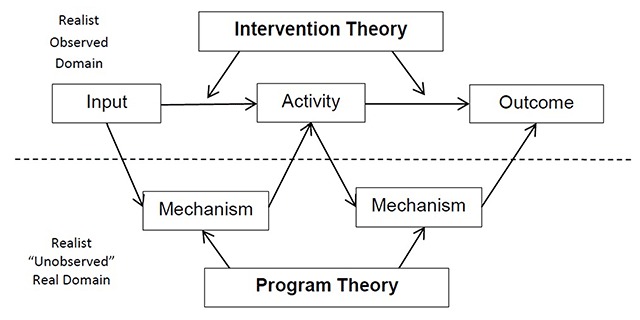
Intervention and Program Theory.

Blamey and Mackenzie [13] propose that the ToC approach be used as a means of explicating [intervention] theory for the purpose of programme planning, improvement and the development of robust monitoring systems at a whole programme level; while realist evaluation approaches be used to examine in detail aspects of the most promising programme (mechanism) theories. In this study protocol we will use both approaches (Figure 3).

### Philosophy of continuous improvement

In keeping with critical realist research methods we propose to use the Realist Evaluation Cycle as proposed by Pawson and Tilley [15]. Critical realism pays particular attention to studying the historical nature of the conditions or context within which the intervention is implemented. Consequently the approach we take will involve base-line critical realist studies that examine the context in the early phase of implementation [16]. This is to enable before and after comparisons to be made so as to establish and track ongoing change being introduced and taking place within the HHAN intervention. Thereafter, the realist evaluation cycle will be used to identify the causal pathways that may explain the outcomes being produced and to surface unexpected outcomes which indicate the need to make further modifications to the way the integrated care initiative is organised. This approach is also in keeping with action research methods and the quality improvement “Plan, Do, Study Act” (PDSA) cyclic approach to adaptive management of programmes. Consequently we will also explore in the following methodology the application of PDSA methodology within a critical realist evaluation approach.

### Protocol Overview

**Figure 4:**
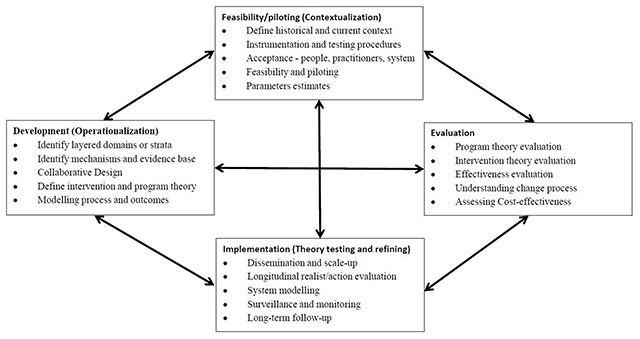
Key elements of the development and evaluation process, adapted from [7].

In summary, we have drawn on the above research and evaluation frameworks and theories to inform our adapted research and evaluation protocol for HHAN as shown in Figure 4.

The double arrows used in the original MRC Framework are intentional and denote the iterative nature of the development, testing, evaluation, implementation cycle. This is not unlike the constant comparative approach used in emergent theory building approaches such as grounded theory. We believe that all elements of the cycle will influence each other throughout the course of the program. Consequently, we have added additional arrows to the model above (Figure 4). The 2008 MRC Framework provided a four-phase, cyclical framework of development, feasibility/piloting, evaluation, and implementation [5]. We have adapted the MRC Framework phases to our previously described operationalisation, contextualisation and evaluation phases as follows:

- Development – Operationalisation
- Feasibility/piloting – Contextualisation
- Evaluation
- Implementation.

The phases, methods, projects and activities are summarised in Table 2.

**Table 2:** Phases, Methods and Proposed projects and activities.

Phase	Methods	Projects/activities

**Development (Operationalization)**	Identifying the layered domains or strata Identifying the mechanisms and evidence base Undertaking collaborative design Defining the intervention and program theory Modelling the process and outcomes	Building realist causal theory [17] Building realist program theory [18] Designing initiatives for vulnerable families [3] Designing HHAN integrated care initiative [2] Systematic literature reviews Meta-narrative and realist synthesis reviews. Building the detailed HHAN Logic Model.
**Feasibility/piloting (Contextualisation)**	Define historical and current context Define instrumentation and testing procedures Assess acceptance by people, practitioners and the system Determine parameter estimates	Delphi study of HHAN context Define the HHAN intervention indicator KPI data set Define and test HHAN patient reported measures Data-linkage studies including GIS and Epidemiology studies Base-line qualitative and mixed method studies of each HHAN intervention component.
**Evaluation**	Program theory evaluation Intervention theory evaluation Effectiveness evaluation Understanding the change process Assessing cost-effectiveness	Realist qualitative and mixed-method HHAN studies, including: Partner-level studies Place-based studies (including practitioners and consumer studies) Quantitative modelling studies of: Patient reported measures Data-linkage studies. Consideration of control designs for clinical component.
**Implementation (Theory Testing and Refining)**	Dissemination and scale-up Longitudinal realist/action evaluation System modelling Surveillance and monitoring Long-term follow-up	Longitudinal HHAN intervention evaluation. Including monitoring of KPIs, system modelling, and ongoing qualitative interviews Longitudinal mixed method study, including HHAN PDSA cycles and monitoring of HHAN PRMs.

### Development (Operationalisation)

The purpose of the Development Phase is to make explicit what we are trying to do, the outcomes we are aiming for and how we intend to bring about change. To achieve this it is essential that the intervention has a coherent theoretical base. In the case of an integrated care intervention the theoretical base will be layered and draw on not only evidence of effective interventions but also effective organisation in the form of a relevant programme, process or mechanistic theory [19]. In the adapted framework we have identified the importance of undertaking collaborative design processes in the Development Phase. The elements are:

1. Identify layered domains or strata
2. Identify mechanisms and evidence base
3. Undertake Collaborative Design
4. Define intervention and program theory
5. Modelling process and outcomes.

Our critical realist methodology for the Development (Operationalisation) Phase has been previously described [8], and we have reported the two design studies that led to the development of this integrated care initiative for vulnerable families [2,3]. As part of that process we specified the layered domains and identified prospective intervention and programme theory from relevant published theories, meta-syntheses and realist synthesis [18]. The resulting Theory of Change and Logic Models have also been previously reported [2].

The Critical Realist research cycle, and both the MRC and NSW Health Evaluation Frameworks, make it clear that the Development Phase is dynamic. Throughout the research and evaluation process the Logic Model will be reviewed. The original two design studies identified a number of evidence-based clinical interventions, including: perinatal psychosocial screening, sustained nurse home visiting, targeted parenting programmes, wrap-around care, and family group conferencing. The integrated care design allowed for other “evidence-based” interventions to be introduced. We propose, therefore, to undertake a number of further Development Phase studies as the intervention is implemented. They will include, but not be limited to: systematic and realist reviews of perinatal psycho-social interventions; universal child and family services; early childhood literacy; parental health literacy; multidisciplinary teams; and place-based child and family initiatives.

The Development (Operationalisation) Phase includes modelling of process and outcomes as part of the development of the Theory of Change and related Logic Models. That analytical process identified the need for measures of context, mechanisms and outcomes. Not all of those measures were available at the time of the design development. Importantly NSW Health had not identified the monitoring and evaluation data sources necessary to assess medium and long-term outcomes. The development of relevant patient and programme indicators is described in the next section.

### Feasibility/piloting (Contextualisation)

The MRC Framework describes the purpose of the feasibility and piloting phase as being to test “procedures for their acceptability, estimating the likely rates of recruitment and retention of subjects, and the calculation of appropriate sample sizes”. Missing from the MRC advice is consideration of the need to study the broader context including: acceptability of the intervention by service providers, and the broader layered service system. Within a layered integrated care intervention the measurement of clinical, process and outcomes indicators is problematic. To enable full piloting to occur it will be necessary to have developed and tested all instrumentation and testing procedures, also giving space to other approaches for evaluating an effect other than using a trial.

In the adapted framework we have identified the importance of defining the context and instrumentation. The modified Feasibility/piloting (Contextualisation) elements are:

- Define historical and current context
- Define instrumentation and testing procedures
- Assess acceptance by people, practitioners, and the system
- Undertake feasibility and piloting
- Determine parameters estimates.

Our critical realist methodology identified the importance of contextualisation of intervention case studies and the subsequent development of data collection tools and approaches in those concrete situations. The integrated care initiative will be evaluated in multiple contexts and system layers. A particular feature of the critical realist approach is the emphasis on studying the full historical and current perspective of the layered context. At a clinical level this involves a comprehensive psychosocial interview. At practitioner, provider and system levels a similar analysis is required. This analysis also has implications for the development of measurement instrumentation.

We have previously observed [8], that it is likely that modifications will be required for interview, focus group, and quantitative instruments to ensure acceptability, appropriateness and validity. For the purposes of our Sydney-based intervention, modifications will be required for data collection from Aboriginal and Torres Strait Islander populations, and those of culturally and linguistically diverse backgrounds. It will also be necessary to modify our data collection approach where domestic violence and severe psychological or physical trauma has been experienced. Given the emergent longitudinal nature of the research we anticipate that the data collection tools will require modification after each analytical cycle.

The Feasibility/piloting (Contextualisation) Phase will include the following studies or bodies of analysis:

- Baseline study of context using a Delphi-style approach
- Indicator development and instrumentation
- Development of person-centered reported measures (aka Patient Reported Measures)
- Data-linkage studies
- Pilot critical realist case studies

#### Delphi Study of Context

The aim of this body of research will be to undertake qualitative studies of barriers and enablers that exist for families that either help or hinder their engagement with services. The method will be a triangulated study consisting of Delphi studies, focus groups and individual semi-structured interviews. Senior staff from partner organisations will be identified by the researchers and contacted to participate in a Delphi-style panel discussion with 8–15 panel members. The aim of the discussion will be to rank the importance of barriers and enablers in the local context. This information will be incorporated into the creation of two separate interview guides; one for frontline staff which will be conducted in the form of a focus group(s), and individual semi-structured interviews with families.

#### Development of Indicator Instrumentation

The purpose of the programme of analysis is to develop relevant indicators of context, programme content, mechanisms and outcomes based on the Programme Theory, ToC and Programme Logic. The indicators and metrics will be developed at individual, family, practitioner, agency and programme level. Individual and family level indicators will be drawn from current clinical policy, practice and various Australian research programmes, such as the Longitudinal Study of Australian Children (LSAC) [20], and Australian Early Childhood Census [21]. Child outcomes indicators, for example, will include: Immunisation status, National Assessment Program – Literacy and Numeracy (NAPLAN) [22], Ages and Stages Questionnaire (ASQ) [23] and Strength and Difficulties Questionnaire (SDQ) [24]. Adult indicators will be drawn from those used in other integrated care initiatives and accepted local clinical practice. The HHAN design identified a number of possible indicators, including: assessments of diabetes, mental health and drug and alcohol use. Practitioner, agency and programme process and outcome indicators will be taken from the Programme Logic Model. Population-level studies will be undertaken using the indicator set and will inform longitudinal spatial-temporal studies of programme impact. The indicator framework will also be used to inform the NSW Health Intervention “Road Map” Evaluation, Person Reported Measures (PRMs), and data-linkage studies (discussed below).

#### Person reported measures (aka PRMs)

The purpose of the PRMs research programme will be to develop and monitor person reported outcome and experience measures for enrolled family members. A PRMs process will be developed using self-reported survey tools that can be administered either during clinical encounters or by access to web or phone-based data entry. A study of suitable measures will be undertaken and trialled during the first operational year. Health and wellbeing measures for both children and adults will be used. The tools will be used for baseline assessment, experience of the programme and self-reported outcomes.

#### Electronic Medical Records (EMR) Data-Linkage Studies

This project aims to develop technical and analytical approaches to the use of routinely collected patient information to examine the impact of implemented integrated care initiatives on the early life experiences and the health, development and welfare of infants born in the Sydney Local Health District (SLHD). This will initially involve linkage and the exploration of routinely collected maternal and child health information sourced from the SLHD EMR databases. The project will undertake both epidemiological (such as examination of association between early life experiences and adulthood health outcomes) and health service research using the linked maternal and child health data. The data will be analysed using Geographical Information System (GIS) methods. Those studies will be used to identifying the geographical distribution of the “most vulnerable” families with intergenerational cycles of disadvantage and trauma in SLHD. The analysis will contribute to identify “hot spots” suitable for the place-based interventions proposed in the intervention design. The EMR programme of research will contribute to the development of population-level measures of impact including studies of hospitalisation, Emergency Department and outpatient attendances by both parents and their children.

#### Baseline Case Studies

The intervention design calls for the progressive implementation of intervention components including: place-based initiatives with wrap-around models of care; family health improvement health literacy projects; general practice and engagement initiatives; strengthened referral pathways; person report measures (PRMs) and system change. For each component of the design, baseline case studies will be undertaken. The case studies will, where appropriate, use critical realist methodology and will seek to understand what is working, for whom and in what circumstances within the family and practitioner strata of the integrated care initiative. Case study methods will be used to explore and examine the context, intervention, mechanisms and outcomes (CIMO), through in-depth qualitative interviews of clients and practitioners, clinical records, and survey tools. Quantitative tools will also be used including: social network analysis, baseline risk assessment, and PRMs (i.e. self-efficacy and quality of life measures). For the place-based projects baseline community consultation will be also undertaken. The findings of these baseline case studies will inform the studies undertaken in the Evaluation and Implementation Phases.

### Evaluation

The complex whole-of-system nature of the HHAN integrated care initiative places significant challenges on the prospect of evaluating the effect and efficacy of the interventions. The 2008 MRC advice proposed the use of: individually randomised trials; cluster randomised trials; stepped wedge designs; randomised consent trials; and N-of-1 designs. Although they focus on evaluating the effect of the intervention, the advice recognised the importance of process evaluation within trials. Moore and colleagues subsequently published MRC-endorsed advice on process evaluation of complex interventions [25] and Richards and Hallberg [26] provided an overview of alternative approaches, including revisiting the use of Bradford-Hill’s thinking on causality. The MRC argues that too many complex interventions are brought to a trial without proper development and feasibility testing, leading to large amount of research waste [27,28]. Although their model is nowadays established worldwide, adding the realist perspective will reduce research waste [29]. The emphasis on evaluating changes in aggregate measures continues to be criticised by realist researchers [30], and consequently Fletcher and colleagues have recently advanced a realist approach to the MRC Framework [31]. We have previously described a critical realist methodology which will be applied here to the evaluation of the HHAN integrated care initiative [8]. That methodology included: mixed method studies; qualitative case studies; quantitative studies, structural equation modelling, and the use of action research and PDSA cycles [32]. The Evaluation Phase will include the following studies or bodies of analysis:

- Realist Mixed-method studies
- Quantitative modelling.

The possibility of including other evaluation designs is currently under consideration, including: nested individual randomised control trials (i.e. targeted parenting initiatives); population-level spatial-temporal analysis; stepped-wedge designs and single-subject designs.

#### Realist Mixed Method studies

The initiatives to be evaluated will be complex with likely multiple contexts and layers as described by Layder [33, p 73]. We anticipate that it will be necessary to focus separate evaluation studies on one level and stage of the logic model (i.e. case-studies). The description of the various contexts will require a full historical and current perspective of the layered context. At the individual client level the contextualisation will entail, for example, a full personal and family history similar to that undertaken in a comprehensive social interview. Where the evaluation is focusing on a situated activity or setting, the documentation is likely to require an exploration of historical pre-existing features of the setting that may themselves be mechanisms with generative power. The methods will be similar to that described above for the Baseline Studies.

Given the nature of the causal and programme theories being investigated we intend to, where possible, focus separate studies on: 1) maternal and family contexts; 2) practitioner contexts; 3) place-based settings; and 4) interagency contexts. The pre-existing vertical relationships in the layered system will also be examined. The Partner-level and Place-based studies will have additional elements.

#### Partner-level studies

These studies aim to understand what is working, for whom and in what circumstances within the implementation of the integrated care initiative at interagency and policy levels. The qualitative component utilises the same methods to those described above for the Realist case studies. The study will explore and examine the context, mechanisms and initiative outcomes (CMO) as experienced by agency and policy participants, through in-depth qualitative interviews, document analysis, and survey tools. The survey tools will include social network analysis and a partnership evaluation tool.

Social network analysis is a method that can be used to identify and map inter-organisational relationships. Interviewees will be asked to complete an online survey in which they nominate other community agencies and organisations with which they collaborated via receipt of referrals, sending referrals to, sharing information about clients, and “working together in other ways.” Respondents can also nominate other agencies not listed in the survey. The nature of those relationships are then described.

#### Place-base case studies

The aim is to understand what is working, for whom and in what circumstances within the Neighbourhood integrated care initiatives. The methods for the place-based case studies are yet to be fully formulated but will include: participatory research methods, realist mixed method studies with families, practitioners and partner agencies (as described above), local General Practice focused studies, and modelling of local quantitative data, including multilevel and spatial studies. The place-based case studies will by necessity include all four phases of the research framework described here. The development, feasibility, evaluation and implementation phases will all be undertaken with local consumer and practitioner input.

#### Quantitative Modelling

Quantitative data will be used to evaluate both programme and intervention theory. Those two purposes are quite distinct with the instruments chosen for programme evaluation being derived from both the causal (MCO) and programme (CIMO) hypotheses developed in the Development (Operationalisation) Phase, and subsequently modified during the intervention evaluation. Given the longitudinal emergent nature of the evaluation it is anticipated that some quantitative measurements will be added or altered during the course of the evaluations. We consider that addition or amendment of quantitative measures enables more valid testing of the middle range theories.

Modelling of quantitative data within a critical realist evaluation is controversial but supported by realist methodologists Sayer [15,34,35,36,37]. We will use the structural modelling approach recently described by Jamal and colleagues [37]. In keeping with earlier realist studies by Kazi [38] the programme evaluations will use previously validated psychometric instruments as measures of hypothesised mechanisms and outcomes. These could include measures of child development and behaviour, self-reported health, self-efficacy, depression, isolation, and health literacy. We have also previously described critical realist approaches to multi-level spatial modelling, factor analysis and regression studies [39,40]. We will use those methods to analyse the data-linkage data collections described earlier.

### Implementation

The MRC Framework provided advice on the implementation of complex interventions with a focus on dissemination of findings, surveillance, monitoring and long-term outcomes. The Framework also stresses the need for changing behaviour of a wide range of people. Understanding the behaviours that need to change, the factors maintaining current behaviour and barriers and facilitators to change is crucial to inform implementation of any initiative that incorporates behaviour change [7]. The NSW Integrated Care Strategy will provide regular forums for the dissemination of findings at each stage of the intervention. The state-wide and local interagency governance structures will also allow for wide dissemination of learnings across the whole-of-government system. Our focus here will be on longitudinal surveillance, monitoring, outcome measurement, and adaptation of the intervention, based on both formative and summative findings. We have consistently identified the need to use realist continuous improvement PDSA cycles and action research approaches to understand historical and current contextual behaviours in the implementation of the HHAN integrated care initiative. This will be achieved by utilising the multi-level critical realist case studies and NSW Health monitoring to continuously assess and modify the intervention.

#### Longitudinal Intervention “Road Map” Evaluation

The aim of the longitudinal intervention evaluation is to develop a Roadmap of Program Milestones (RPM) based on key evaluation questions emerging from the logic map and functional components of the design. The NSW Health Integrated Care Strategy: Monitoring and Evaluation Framework [4] proposed the use of both qualitative and quantitative indicators, to allow assessment of actual outcomes relative to expected outcomes. The RPM will be used to identify whether the program is ‘on track’. Key milestones will be identified that indicate logical progress towards the intended outcomes of the program. The logic mapping allows the key annual milestones to be purposefully identified, and to be updated at the beginning of each year. Analysis of the RPM will be used to support other evaluation components including the critical realist, outcome and enabler studies. The longitudinal study will include regular qualitative interviews with programme managers and stakeholders regarding progress against an agreed set of qualitative indicators that are based on the functional components. The programme stakeholders will also be regularly involved in programme review workshops.

#### Longitudinal Critical Realist Studies

The aim of the longitudinal critical realist study is to understand what is working, for whom and in what circumstances within the implementation of the integrated care initiative at family, practitioner and agency levels. A longitudinal emergent realist mixed method study design will be used. For the qualitative component, the same methods to those described above for the Realist case studies will be used. The findings of the individual case studies will be aggregated [15].

The Program Theory will be modified based on aggregated findings. Based on the modified Program Theory, semi-structured interview schedules will be developed to further examine proposed mechanisms. The above will be complemented by client and practitioner focus groups to further examine the mechanisms proposed to be operating within the individual family – practitioner configurations. The practitioner and agency CMO configurations will be examined utilising practitioner and agency level interviews and focus groups.

## Discussion

When designing the Healthy Homes and Neighbourhoods Initiative we built on a previous programme of mixed method multi-level critical realist empirical research and theory building. The challenge of integrating care for families under stress necessitated a complex design. We have addressed this through adapting and incorporating relevant evaluation frameworks, theories and quality improvement methodologies to inform our adapted conceptual framework for the evaluation of HHAN. To do justice to the complexity of the context, several research methodologies have been integrated in this paper. This may seem like a methodological paper, but the application of these methodologies to the complex context of the HHAN program is an essential part of our message in this paper. The consequence of undertaking research in relation to integrated care is that the complexity of the research, and the methodologies required, will increase in relation to that usually applied for singular interventions.

Both the 2008 UK Medical Research Council Framework for evaluating complex interventions, and our previously reported critical realist methodology, provided the tools to enable an iterative approach development, design, testing and continuous evaluation. The reporting requirements of the NSW Health Integrated Care Strategy: Monitoring and Evaluation Framework provided an additional challenge as did the requirement to use Theory of Change approaches together with a realist methodology.

As discussed earlier, the philosophical paradigms underpinning each approach are different. Both the NSW Health and MRC Frameworks were developed from the “logical positivist” inductive and deductive reasoning traditions with a strong focus on activities and outcomes but little emphasis on process. Fortunately this weakness has been recognised and Moore and colleagues subsequently published MRC-endorsed advice on process evaluation of complex interventions [25].

The use of the term “implementation” in the MRC Framework is problematic as both the NSW Health Monitoring and Evaluation Framework and Theory of Change terminology use the term “implementation” with different meaning. To avoid confusion we have used the term “intervention” where those two frameworks would have used “implementation”. The MRC Framework is primarily focused on the evaluation of efficacy and effectiveness of intervention “content”. By contrast integrated care and realist evaluation approaches are predominantly about process and mechanisms in context respectively. As noted elsewhere those two perspectives can be reconciled with the inclusion of process and realist orientated research methods [25,41] as demonstrated in this framework.

As observed earlier, similar challenges exist when using the Theory of Change approach to evaluation. We have drawn here on the work of Blamey and Mackenzie [13] who compared Theories of Change and Realist approaches to evaluation. Blamey and Mackenzie [13], proposed that the Theory of Change approach be used as a means of explicating [intervention] theory for the purpose of programme planning, improvement and the development of robust monitoring systems at a macro programme level; while realist evaluation approaches be used to examine micro level aspects of the most promising programme (mechanism) theories.

In program implementation and evaluation, these ontological tenets have implications on how the accounts of integrated care service delivery, and the claims made about their impact, are to be assessed. In this program of research and evaluation, possible threats to validity arising from the accounts developed will be addressed using criteria proposed by realists [42] who view ‘the validity of an account as inherent, not in the procedures used to produce and validate it, but in its relationship to those things that it is intended to be an account of’. The kinds of threats to validity that the HHAN initiative aims to address in practice are those associated with descriptive, interpretive, theoretical, generalisability and evaluative validity as elaborated by Maxwell [42]. A full exploration of realist approaches to validity is not possible here, but it is our intention to ensure each protocol developed under this methodological framework, makes clear the ontological approach to validity that is being used.

## Conclusion

Integrated care initiatives are examples of complex interventions in health and social care. The interventions are multi-layered and operationalisation is strongly influenced by both historical and current context. This aspect alone makes it imperative that the research and evaluation methodology used is post-positive and takes ontologically stratified context into account. The NSW Health Monitoring and Evaluation Framework did not make provision for assessment of context or mechanism of effect. We describe here a multilevel approach, including a continuous improvement approach, through constant comparison and triangulation of mixed method findings. Finally the research and evaluation protocol described here, has utlised the MRC Framework for evaluating complex interventions within a critical realist methodology, thus enabling us to study both mechanisms of effect and context. As such the innovative methodology utilised here is of potential relevance to other researchers facing similar challenges in evaluating integrated care.
